# The Recruitment of AMP-activated Protein Kinase to Glycogen Is Regulated by Autophosphorylation*

**DOI:** 10.1074/jbc.M114.633271

**Published:** 2015-03-19

**Authors:** Yvonne Oligschlaeger, Marie Miglianico, Dipanjan Chanda, Roland Scholz, Ramon F. Thali, Roland Tuerk, David I. Stapleton, Paul R. Gooley, Dietbert Neumann

**Affiliations:** From the ‡ Department of Molecular Genetics, CARIM School of Cardiovascular Diseases, Maastricht University, 6200 MD Maastricht, The Netherlands,; the § Institute of Cell Biology, ETH Zurich, 8093 Zurich, Switzerland, and; the ¶ Florey Institute of Neuroscience and Mental Health and; ‖ Department of Biochemistry and Molecular Biology, Bio21 Molecular Science and Biotechnology Institute, University of Melbourne, Victoria 3010, Australia

**Keywords:** AMP-activated Kinase (AMPK), Carbohydrate-binding Protein, Cellular Regulation, Glycogen, Phosphorylation, Post-translational Modification (PTM), Autophosphorylation, Subcellular Localization

## Abstract

The mammalian AMP-activated protein kinase (AMPK) is an obligatory αβγ heterotrimeric complex carrying a carbohydrate-binding module (CBM) in the β-subunit (AMPKβ) capable of attaching AMPK to glycogen. Nonetheless, AMPK localizes at many different cellular compartments, implying the existence of mechanisms that prevent AMPK from glycogen binding. Cell-free carbohydrate binding assays revealed that AMPK autophosphorylation abolished its carbohydrate-binding capacity. X-ray structural data of the CBM displays the central positioning of threonine 148 within the binding pocket. Substitution of Thr-148 for a phospho-mimicking aspartate (T148D) prevents AMPK from binding to carbohydrate. Overexpression of isolated CBM or β1-containing AMPK in cellular models revealed that wild type (WT) localizes to glycogen particles, whereas T148D shows a diffuse pattern. Pharmacological AMPK activation and glycogen degradation by glucose deprivation but not forskolin enhanced cellular Thr-148 phosphorylation. Cellular glycogen content was higher if pharmacological AMPK activation was combined with overexpression of T148D mutant relative to WT AMPK. In summary, these data show that glycogen-binding capacity of AMPKβ is regulated by Thr-148 autophosphorylation with likely implications in the regulation of glycogen turnover. The findings further raise the possibility of regulated carbohydrate-binding function in a wider variety of CBM-containing proteins.

## Teaser

**Background:** AMP-activated protein kinase (AMPK) is a current drug target. AMPK can attach to glycogen granules.

**Results:** Autophosphorylation of AMPK prevents its association with glycogen.

**Conclusion:** Subcellular localization of AMPK is affected by the kinase autophosphorylation status.

**Significance:** Understanding the regulation of AMPK at subcellular level is crucial for the currently pursued drug targeting approach.

## Introduction

Many enzymes involved in the synthesis and degradation of complex carbohydrate molecules, such as cellulose or glycogen, contain a domain called the carbohydrate-binding module (CBM)^4^ specialized in the binding of complex carbohydrates. CBMs are present in various taxa and protein types (1, 2). Despite their lack of direct catalytic activity, CBMs may enhance specific enzymatic activity by targeting the enzyme to its substrates and increasing its effective concentration (3, 4). Other proteins that are not directly carbohydrate-active but are involved in glucose metabolism regulation are also known to contain a CBM; this is notably the case for the mammalian energy sensor AMP-activated protein kinase (AMPK) (5, 6) as well as its fungal homolog SNF1 (7) and its plant homolog SnRK1 (8).

AMPK is a heterotrimer consisting of (i) a catalytic subunit α (α1 or α2) carrying the kinase domain, (ii) a regulatory subunit β (β1 or β2) with the CBM and the C-terminal region tethering α and γ together, and (iii) a regulatory subunit γ (γ1, γ2, or γ3) responsible for the adenine nucleotide sensing (9, 10). AMPK is a well known, highly conserved metabolic enzyme central for coordinating cellular and whole body energy homeostasis. Upon cellular stress, such as contraction, exercise, or hypoxia, AMPK is activated by several mechanisms. Briefly, the increased AMP level induces a conformational change in AMPK facilitating the phosphorylation of Thr-172 on the activation loop of the α-subunit by upstream kinases, such as liver kinase B1 (LKB1) or calcium/calmodulin-dependent protein kinase 2 (CaMKK2), resulting in roughly 1000-fold activation of the enzyme (11–13). The AMP-binding to AMPKγ also reduces the rate of dephosphorylation at Thr-172, thus keeping AMPK in its active state (11, 14). As a result, AMPK phosphorylates a number of downstream targets to restore energy balance by facilitating glucose uptake, glycolysis, and fatty acid oxidation, thus increasing the energy production and simultaneously switching off ATP-consuming pathways, such as glycogen, fatty acid, and cholesterol synthesis (9, 12).

Considering the micromolar affinity of recombinant AMPK complexes and CBM for small cyclic and linear oligosaccharides (15, 16), the interaction between AMPK and glycogen in cells may be relatively strong. Because some AMPK targets, such as glycogen synthase (17), are associated with glycogen (18), the presence of a CBM might be beneficial in juxtaposing the kinase next to its substrates (5). However, in the case of targets not located at glycogen, such as the acetyl-CoA carboxylase 2, which is associated with the mitochondria (19), the retention of AMPK at glycogen by the CBM could be detrimental for a quick cellular response to energy stress. In addition, AMPK has been detected in different subcellular areas, for example at the cell membrane (20, 21) or in the nucleus (22, 23). This variety of localizations and actions implies that the AMPK-carbohydrate-binding ability may be regulated in cells. In this study, using cell-free and cellular systems, we show that AMPK loses its carbohydrate-binding ability upon activation due to a bimolecular autophosphorylation event that modifies the AMPKβ-CBM at Thr-148.

## EXPERIMENTAL PROCEDURES

### 

#### 

##### Plasmids

Bacterial expression plasmids encoding for hexahistidine-tagged wild type (WT) AMPK (α1β1γ1, α1β2γ1, α2β1γ1, α2β2γ1) (24, 25), non-tagged LKB1-MO25-STRADα complex (26), and GST-tagged CaMKK2 (11) have been described. The isolated β1- and β2-CBMs were bacterially expressed as described (16). The cDNA of β1 lacking the CBM (amino acids 182–270) was amplified by PCR and initially subcloned into the NcoI and SpeI restriction sites of the pET3dx vector (24). Subsequently, the bacterial expression plasmid encoding for the heterotrimeric hexahistidine-tagged AMPK lacking the CBM (α1β1ΔCBMγ1) was generated using the published procedure (24). The kinase-deficient AMPKα1 plasmids with a non-phosphorylatable alanine (D157A) (α1β1γ1-KD or α1β2γ1-KD, respectively) were constructed as described earlier (27). The coding sequence for Thr-148 in the AMPKβ1 subunits was changed to encode a non-phosphorylatable alanine (T148A) or a phosphorylation-mimicking aspartate (T148D) by site-directed mutagenesis following the manufacturer's instructions (QuikChange, Stratagene).

To obtain activated AMPK phosphorylated at Thr-172, each of the tricistronic WT and kinase-deficient coding sequences were combined with the LKB1-MO25-STRADα coding sequence in a single hexacistronic plasmid, allowing for co-expression of both protein complexes and the subsequent purification of the hexahistidine-tagged AMPK alone (referred to as α1β1ΔCBMγ1 active, α1β1γ1 and α1β2γ1 active, α1β1γ1-KD pT172, and T148A active, respectively) as also described recently (28).

For expression in mammalian cells, the cDNA of WT β1-CBM (amino acids 68–163) or β2-CBM (amino acids 67–163) was amplified by PCR and ligated in frame into the pAcGFP expression vector (Clontech) via the EcoRI and SalI restriction sites, resulting in expression constructs for β1-CBM-WT-GFP or β2-CBM-WT-GFP, respectively. The cDNA of full-length β1 was amplified by PCR and subcloned either into the pmCherry expression vector (Clontech) via EcoRI and SalI restriction sites or into the pCMV5–3xHA expression vector (29) via HindIII and SalI restriction sites. GFP-tagged CBMs or mCherry-tagged β1 bearing a threonine-to-aspartate mutation on residue 148 (T148D) was generated using the QuikChange site-directed mutagenesis kit (Stratagene). The pcDNA3 constructs for the expression of AMPKα1-Myc and γ1-subunit were kindly provided by Dr. D. Carling (Imperial College London).

For retroviral overexpression, GFP-tagged β1-CBM or β2-CBM (either WT or T148D) was subcloned, using an oligonucleotide linker, into the EcoRI and SalI restriction sites of the pBMZ-ires-neo retroviral backbone (kindly provided by Dr. G. Nolan, Stanford University). Primer sequences are available upon request. All of the constructs were verified by sequencing.

##### Bacterial Expression and Purification

Proteins were expressed in Rosetta 2 (DE3) *Escherichia coli* cells (Novagen). Bacteria were grown in autoinduction medium, and proteins were purified as described with few modifications (30). Briefly, the bacterial pellet was collected by centrifugation, resuspended in lysis buffer (for hexahistidine-tagged proteins, 50 mm NaH_2_PO_4_, 30% glycerol, 0.5 m sucrose, 10 mm imidazole, pH 8; for GST-tagged proteins, PBS, pH 7.3) and lysed using a high pressure homogenizer. After centrifugation, the supernatant was incubated for 1 h at 4 °C on a roller bank in the presence of either 1 ml of nickel-Sepharose HP (GE Healthcare) for the hexahistidine-tagged proteins or 1 ml of glutathione-Sepharose 4B (GE Healthcare) for the GST-tagged proteins. After centrifugation, the resin was washed three times with wash buffer (for hexahistidine-tagged proteins, 50 mm NaH_2_PO_4_, 30% glycerol, 0.5 m sucrose, 20 mm imidazole, pH 8; for GST-tagged proteins, PBS, pH 7.3), and then the protein was eluted in elution buffer (for hexahistidine-tagged proteins, 50 mm NaH_2_PO_4_, 30% glycerol, 0.5 m sucrose, 250 mm imidazole, pH 8; for GST-tagged proteins, 50 mm Tris-HCl, 10 mm reduced glutathione, pH 8), frozen in liquid nitrogen, and kept at −80 °C until use.

##### β-Cyclodextrin Binding Assay

β-Cyclodextrin was immobilized on epoxy-activated Sepharose 6B (GE Healthcare) according to the manufacturer's protocol. Briefly, Ni^2+^-affinity-purified AMPK was rebuffered into assay buffer (10 mm Tris-HCl, pH 7.2) using PD-10 columns (GE Healthcare), and 500 μl (25 μg/ml) of this AMPK solution (I fraction) was incubated with 50 μl of pre-equilibrated β-cyclodextrin resin in a 0.7-ml reaction tube (LoBind, Eppendorf) with gentle agitation for 15–20 min. The supernatant containing the non-bound material (S fraction) was removed after centrifugation (2000 × *g*, 1 min) and the resin was washed twice in assay buffer. The wash fractions were discarded. Bound AMPK was eluted by incubation in 500 μl of elution buffer (5 mm β-cyclodextrin in assay buffer) for 5 min with gentle agitation. After centrifugation the supernatant (P fraction) was collected, the resin was washed twice with elution buffer, and supernatants were discarded. Finally, the resin was incubated with SDS-sample buffer at 95 °C to obtain the precipitated fraction (L fraction). For analyses, 5 μl of each fraction were subjected to SDS-PAGE and Western blotting and further processed with AMPKα and phospho-AMPK (Thr(P)-172) antibodies (Cell Signaling Technologies).

##### Phosphorylation Assay of Recombinant Proteins

AMPK was activated *in vitro* by upstream kinases as described previously (30). Briefly, recombinant AMPK (α1β1γ1 or α1β2γ1, 25 μg/ml) was activated in kinase buffer by recombinant GST-CamKK2 (15 μg/ml) for 30 min at 37 °C and subsequently incubated with β-cyclodextrin resin for 10 min with gentle agitation to allow for binding. Alternatively, recombinant AMPK was first incubated with β-cyclodextrin resin, followed by activation with upstream kinases. As described above, the assay was continued with three washing steps and then three times β-cyclodextrin elution buffer and finally SDS-sample buffer. Subsequently, the presence of (phosphorylated) AMPK in the various fractions (I, S, P, and L) was probed by Western blot analysis.

##### Cell Culture

The human hepatocyte HepG2 and human embryonic kidney 293T cell line (HEK293T) were cultured in DMEM with high glucose (25 mm) (Gibco), supplemented with 10% (v/v) heat-inactivated fetal calf serum (Bodinco BV, Alkmaar, The Netherlands) and penicillin/streptomycin (Invitrogen), unless otherwise stated. HL-1 cardiomyocyte cell line was kindly provided by Dr. W. Claycomb (Louisiana State University, New Orleans, LA), cultured on fibronectin (5 μg/ml; Sigma)/gelatin (0.01%; Merck) coating in Claycomb medium (supplemented with 10% heat-inactivated fetal calf serum (iFCS), 0.1 mmol/liter noradrenaline, 2 mmol/liter l-glutamine, 100 units/ml penicillin, and 100 g/ml streptomycin) at 37 °C and 5% CO_2_.

For transient transfections, HEK293T cells were seeded to 30% confluence in 6-well plates (Greiner Bio-one) 24 h before transfection. Cells were co-transfected with plasmid DNA (α1-Myc, mCherry-tagged β1-WT/β1-T148D, and γ1 for immunoprecipitation and Western blotting, or α1-Myc, HA-tagged β1, γ1, and mCherry-tagged β1-WT/β1-T148D for localization studies of the holoenzyme) using Lipofectamine 2000 (Invitrogen) in antibiotic-free culture medium. Six to eight hours after transfection, transfection medium was replaced by normal growth medium. At 24–48 h after transfection, cells were either harvested or fixed.

For the glycogen depletion experiments, cells were either maintained in high glucose medium (DMEM with 25 mm glucose and 10% iFCS) or treated with forskolin (100 μm; Sigma) in the same medium or glucose-deprived (glucose-free DMEM, 10% iFCS) for 16 h.

In order to activate cellular AMPK, cells were serum-starved (plain DMEM, 5.5 mm glucose) for 16 h and subsequently treated with 5-aminoimidazole-4-carboxamide riboside (AICAR, 1 or 1.5 mm; Sigma), oligomycin (3 or 5 μm; Sigma), A769662 (100 μm), phenformin (1.5 mm), vehicle (DMSO), or high glucose medium for 45–60 min. Insulin (100 nm; Sigma) treatment for 15 min was done in order to stimulate glycogen synthesis.

##### Immunoprecipitation and Immunoblotting

Cells were lysed in immunoprecipitation lysis buffer (20 mm Tris-HCl, pH 8.0, 137 mm NaCl, 10% glycerol, 1% Triton X-100, 2 mm EDTA), supplemented with protease and phosphatase inhibitor mixtures (Roche Applied Science). Endogenous AMPK was immunoprecipitated using a combination of AMPKα1 and AMPKα2 antibodies raised in sheep (kindly provided by G. Hardie). Myc-tagged AMPK was immunoprecipitated using a Myc tag antibody (Cell Signaling Technology, Beverly, MA). The primary antibody was incubated top-over-top with 350 μg of protein lysate at 4 °C for 16 h, followed by incubation with protein G-Sepharose beads for 3–4 h at 4 °C. The immune complexes were then collected by centrifugation. The immunoprecipitated proteins were electrophoresed by SDS-PAGE and analyzed by Western blot analysis. Immunoblot analysis was carried out with the following primary antibodies: Myc tag, AMPKα, phospho-AMPK-Thr-172, Akt, phospho-Akt-Ser-473, and GS (all from Cell Signaling). GS-Ser(P)-7 antibodies were a kind gift from G. Hardie. In order to detect changes in Thr-148 phosphorylation, a phospho-specific AMPKβ-Thr-148 antibody was produced (peptide sequence AMPKβ2(142–154) Thr(P)-148, VTSQLGINNLI) (5). Detection was performed using anti-rabbit or anti-mouse horseradish peroxidase (HRP)-conjugated secondary antibodies (Cell Signaling Technology or Dako, respectively), followed by chemiluminescence.

##### Biochemical Cellular Glycogen Measurement

Extraction of glycogen from 293T cells was adapted from a method described by McMahon and Frost (31). Briefly, cells were lysed in potassium hydroxide (30%) and boiled at 70 °C for 30 min. Subsequently, samples were cooled to 25 °C before sodium sulfate (6%, w/v) and EtOH (99.5%, v/v) were added at a 1:1:3 ratio. After thorough mixing, samples were rotated top-over-top at 4 °C for 30–60 min. The precipitate was collected by centrifugation at 5000 rpm for 5 min at 4 °C. To hydrolyze, pellets were dissolved in 1 m HCl and boiled at 100 °C for 2 h. Samples were cooled before neutralization using 2 m NaOH. Hydrolysates were used for glucose determination using a glucose (GO) assay kit (Sigma), according to the manufacturer's instructions.

##### Retroviral Vectors and Infections

Retroviral systems and Phoenix helper-free retrovirus producer cell lines were used as described (32–34). Amphotropic retroviral supernatants were produced following calcium phosphate/DNA transfection of producer cells; 24–48 h post-transfection, the supernatants were harvested, filtered (0.45-μm filters; Corning, Germany), and used for infection of HepG2, HEK293T, and HL-1 cells in the presence of 4 μg/ml Polybrene (Sigma). For infections, cells were incubated with virus particles for 6–8 h and then allowed to recover for 48 h on fresh medium before selection pressure was applied. Infected cells were selected with 200–500 μg/ml G418 (PAA Laboratories GmbH) for 2 weeks preceding experiments.

##### Immunocytochemistry

Cells were grown in 12-well plates (Greiner Bio-One) on coverslips (Ø 20 mm; Thermo Scientific) to 60–80% confluence. Cells were washed twice with PBS and fixed with 4% formaldehyde in PBS for 10 min at room temperature. Fixed cells were stored at 4 °C in PBS-NaN_3_ (0.03%) or washed three times with PBS+/+ (Gibco) and directly used for immunocytochemistry. Subsequently, cells were permeabilized (0.1% Triton X-100 and 0.2% BSA in PBS) for 15 min and blocked (2% BSA-PBS) for 30 min at room temperature. Primary and secondary antibodies (2% BSA-PBS) were incubated for 1 h at room temperature. Coverslips were washed and mounted onto glass slides using DABCO-glycerol medium (Sigma-Aldrich) containing DAPI (1:10,000; Sigma-Aldrich) in order to counterstain nuclei. The anti-glycogen antibody (1:500) was courtesy of Dr. O. Baba (Tokyo Medical and Dental University, Tokyo, Japan), and the secondary antibody was goat anti-mouse IgM Alexa647 (1:200; Invitrogen) or goat anti-mouse IgM Alexa488 (1:200; Invitrogen).

##### Microscopy and Image Capturing

Fixed cells were imaged using a Leica TCS SPE confocal laser scanning microscope (Leica Microsystems GmbH) equipped with an air-cooled argon-krypton mixed gas laser, using oil immersion objectives (×63, numerical aperture = 1.4). Optical sections were recorded with three scans for each image. ImageJ software was used to process and analyze the images. Image brightness and contrast was adjusted to the same settings where needed.

##### Statistical Analysis

All bar graph data are presented as means ± S.E. Statistical analysis was performed by using Student's *t* test and statistical analysis software Prism version 4 (GraphPad Software, Inc.). A *p* value of <0.05 was considered statistically significant.

## RESULTS

### 

#### 

##### The Carbohydrate-binding Ability of AMPK Is Lost upon Activation

**FIGURE 1.**
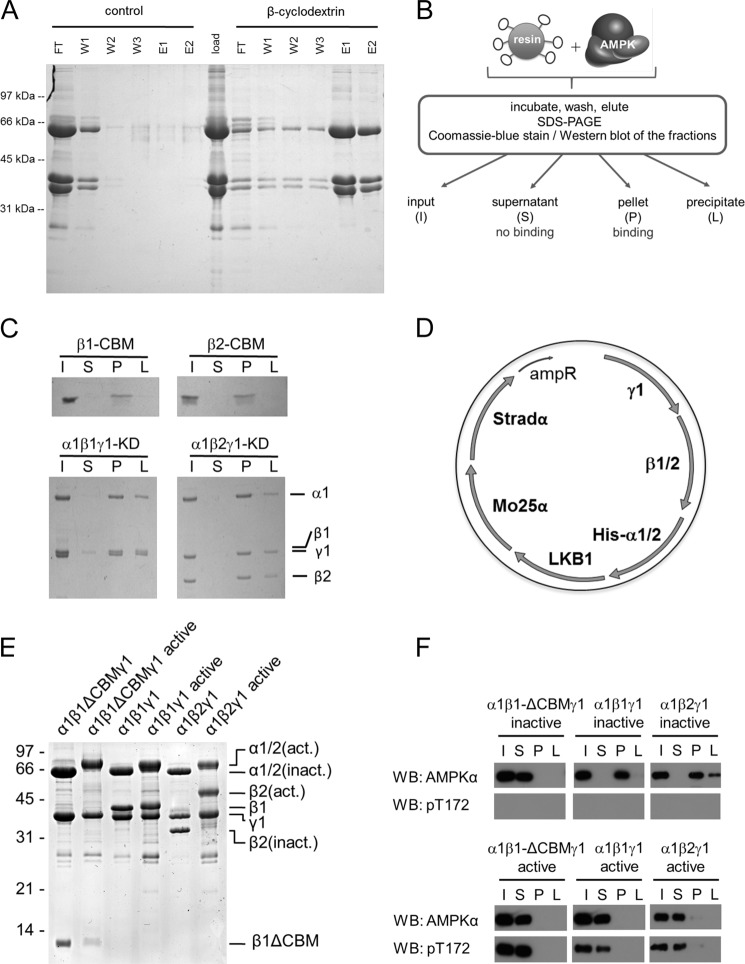
**The carbohydrate-binding ability of AMPK is lost upon activation.** *A*, application of immobilized β-cyclodextrin for purification of AMPK. Epoxy-activated Sepharose was allowed to react with soluble β-cyclodextrin or was inactivated (control). The column resin was equilibrated in 100 mm Tris-HCl, pH 7.2 (binding buffer). Bacterially expressed AMPK heterotrimers (α1β1γ1) were Ni^2+^-affinity-purified. 4.6 mg of the eluate was subsequently rebuffered to binding buffer. The AMPK sample was split into halves and loaded to either control resin or resin with immobilized β-cyclodextrin (500-μl bed volume each). The flow-through (*FT*) was collected, and both columns were washed repeatedly with binding buffer (*W1–W3*) and then eluted twice with binding buffer containing 5 mm β-cyclodextrin. 10 μl of each fraction was subjected to SDS-PAGE, and the gel was stained by Coomassie Brilliant Blue. *B*, schematic model illustrating the β-cyclodextrin binding assay protocol. *C*, His-tagged CBM (β1-CBM or β2-CBM) and kinase-deficient AMPK (α1β1γ1-KD or α1β2γ1-KD) were subjected to the β-cyclodextrin binding assay and visualized by Coomassie Brilliant Blue stain. *D*, schematic representation of the hexacistronic expression vector containing both AMPK (*e.g.* α1β1γ1) and its upstream kinase LKB1-MO25α-Stradα, allowing for “*in vivo*” activation of AMPK in the bacterial cytosol and subsequent purification of active hexahistidine-tagged AMPK (28). *E*, detection of purified inactive and active heterotrimeric AMPK (α1β1γ1 or α1β2γ1) and AMPK complexes lacking the CBM (α1β-ΔCBMγ1) by Coomassie Brilliant Blue stain. Recombinant active proteins were bacterially expressed by means of hexacistronic plasmids. *F*, immunoblot analysis of inactive and active (Thr(P)-172) WT α1β1γ1/α1β2γ1 or truncated AMPK complexes (α1β1ΔCBMγ1). AMPK binding to β-cyclodextrin was assessed using the total AMPKα antibody. Activation of AMPK was assessed using the Thr(P)-172 antibody. Data are representative of three experiments. *WB*, Western blot.

To verify the carbohydrate-binding ability of AMPK, β-cyclodextrin was coupled to Sepharose beads, and recombinant AMPK (α1β1γ1) was expressed in bacteria, purified, and loaded onto the packed column. As shown in Fig. 1*A*, immobilized β-cyclodextrin retains the AMPK complex in the column and could therefore be used to affinity-purify recombinant AMPK. On the contrary, when no β-cyclodextrin was immobilized to the Sepharose, the overexpressed AMPK was not retained, confirming that AMPK retention is dependent on β-cyclodextrin. In further experiments, this procedure was simplified by allowing the recombinant AMPK to bind to the β-cyclodextrin resin in a batch set-up (Fig. 1*B*) instead of using a column. Fractions of the protein that could not bind to the resin stayed in the supernatant (S), whereas fractions that bound to the resin were eluted from this initial pellet fraction (P) by inclusion of soluble β-cyclodextrin in the buffer. The remaining resin-bound proteins were solubilized by extraction with Laemmli buffer (L) and considered as nonspecific precipitates. All fractions were collected and analyzed by SDS-PAGE followed by Coomassie Brilliant Blue stain or Western blotting. This assay was performed both with the isolated CBM (β1-CBM or β2-CBM, respectively) and a kinase-dead (KD) mutant of AMPK (α1β1γ1-KD or α1β2γ1-KD, respectively) (Fig. 1*C*). As expected, both CBM isoforms as well as both catalytically inactive AMPK complexes were able to bind to the resin and were consequently found in the pellet fraction.

To obtain Thr-172-phosphorylated active AMPK, the tricistronic plasmid for AMPK expression (24, 25) was combined with the LKB1-Mo25α-Stradα tricistron, resulting in a hexacistronic AMPK-LKB1 co-expression construct (Fig. 1*D*). AMPKα is the only protein of the plasmid constructs carrying a His tag that resulted in purified AMPK complexes when using the His tag purification protocol. Heterotrimeric α1β1γ1 and α1β2γ1 AMPK complexes, as well as the AMPK α1β1γ1 complex lacking the N-terminal CBM (α1β1-ΔCBMγ1), were expressed and purified in their inactive and active state (Fig. 1*E*). As shown in Fig. 1*F*, the α1β1-ΔCBMγ1 complex was expectedly found in the supernatant fraction and therefore did not retain the ability to bind to carbohydrates (Fig. 1*F*, *top*). As seen before with the KD mutants, the inactive forms of the AMPK complexes containing full-length β1 or β2 normally bound to β-cyclodextrin because these were found in the pellet fraction. In contrast, the active AMPK isoforms showed a loss of binding affinity to the β-cyclodextrin resin similar to the truncated AMPK complex (Fig. 1*F*, *bottom*). These findings suggest that activation of AMPK prevents its later binding to carbohydrates, such as β-cyclodextrin.

##### AMPK Activation by Upstream Kinases Is Not Directly Responsible for the Loss of Binding Ability to Carbohydrates

**FIGURE 2.**
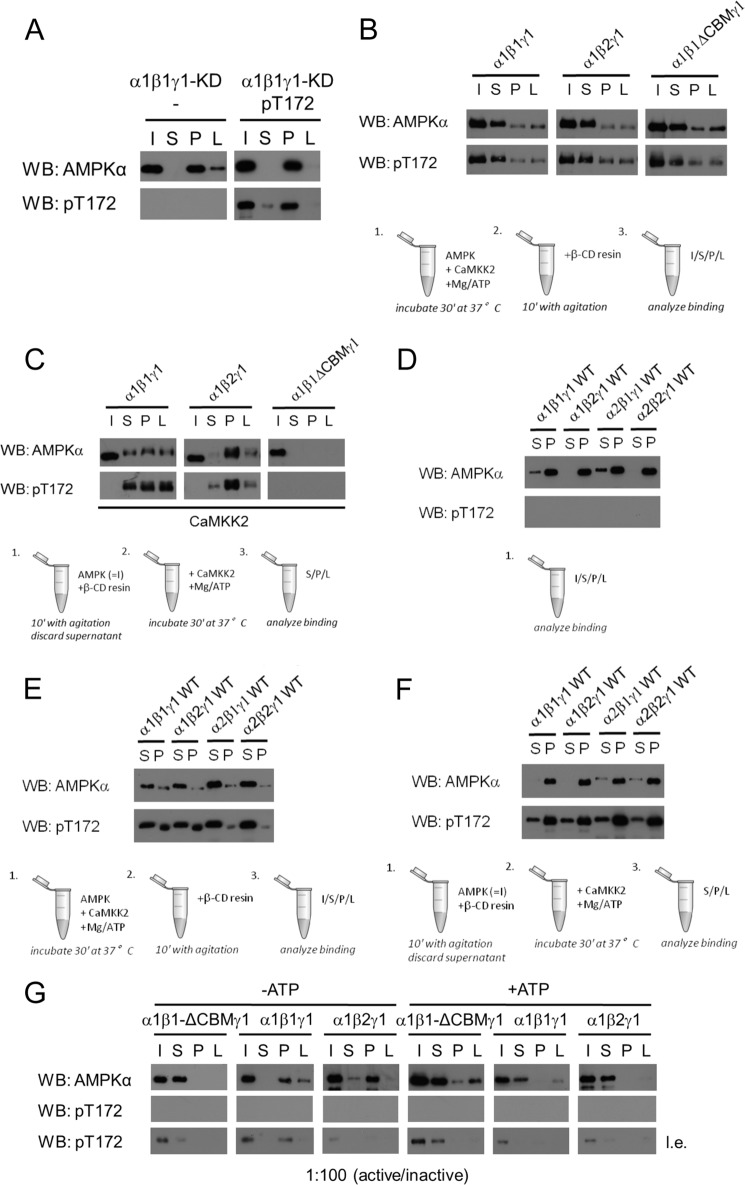
**AMPK activation by upstream kinases is not directly responsible for the loss of binding ability to carbohydrates.** *A*, immunoblot of non-phosphorylated and phosphorylated (Thr(P)-172) recombinant kinase-deficient AMPK (α1β1γ1-KD) (as in Fig. 1) subjected to the β-cyclodextrin binding assay. *B* and *C*, immunoblot of WT (α1β1γ1 or α1β2γ1) or truncated recombinant AMPK complexes (α1β1ΔCBMγ1) (as in Fig. 1) phosphorylated *in vitro* by the upstream kinase CaMKK2 prior to exposure to β-cyclodextrin (*B*) or after initial binding to β-cyclodextrin (*C*). *D–F*, four different isoforms of AMPK heterotrimers were subjected to the β-cyclodextrin binding assay. Shown are immunoblot analysis of inactive AMPK complexes (*D*), AMPK complexes that were *in vitro* phosphorylated by the upstream kinase CaMKK2 prior to β-cyclodextrin exposure (*E*), and AMPK complexes that were *in vitro* phosphorylated after initial binding to β-cyclodextrin (*F*). *G*, inactive and active AMPK complexes were mixed (100:1 ratio) and incubated in the presence or absence of ATP and subjected to the β-cyclodextrin binding assay. In all experiments, the effect on AMPK binding to the model carbohydrate was evaluated. AMPK binding and activation status of AMPK was assessed using the total AMPKα and Thr(P)-172 antibodies, respectively. Data are representative of three experiments. *l.e.*, long exposure; β-*CD*, β-cyclodextrin; WB, Western blot.

To test whether phosphorylation of Thr-172 in AMPKα was sufficient to cause the loss of carbohydrate-binding, a KD mutant of AMPK α1β1γ1 was bacterially expressed in the presence or absence of the LKB1-Mo25α-Stradα complex and subsequently purified. In contrast to WT AMPK, both Thr-172-phosphorylated and non-phosphorylated KD complexes were found in the pellet fraction, indicating that catalytically inactive AMPK did not lose its ability to bind to the β-cyclodextrin when co-expressed with the LKB1-Mo25α-Stradα complex (Fig. 2*A*). Thus, phosphorylation of AMPK at Thr-172 by an upstream kinase is insufficient to trigger the loss of carbohydrate binding, suggesting that AMPK enzyme activity is required.

To confirm this, AMPK α1β1γ1, α1β2γ1, and α1β1-ΔCBMγ1 were expressed as inactive kinases (*i.e.* without co-expression of the LKB1-Mo25-Stradα complex) for subsequent activation *in vitro* before or during the β-cyclodextrin binding assay. Bacterial expression of recombinant CaMKK2 yields higher purity than LKB1 complex and was therefore used as an alternative upstream kinase of AMPK for these assays. As shown by the phosphorylation of Thr-172, all AMPK isoforms were activated and, as expected, mostly lost their binding ability to the resin material (Fig. 2*B*), indicating that activation of AMPK leads to loss of its carbohydrate-binding ability irrespective of the identity of the upstream kinase used, CaMKK2 or LKB1. However, when inactive AMPK was initially allowed to bind to the β-cyclodextrin resin and was subsequently incubated with CaMKK2, both AMPK isoforms retained their ability to bind to the resin because the proteins were predominantly found in the pellet fraction, despite phosphorylation at Thr-172 (Fig. 2*C*). The β2-isoform showed stronger retention upon activation compared with the β1 complex, which corresponds to a higher binding affinity of the β2-CBM for β-cyclodextrin (16). In this experimental setup, the α1β1-ΔCBMγ1 only appears in the input and not in the S or P fraction due to its inability to bind the β-cyclodextrin resin, and therefore the protein is lost upon removal of the supernatant in step 1. Similar results were obtained using α2-containing (α2β1γ1 and α2β2γ1) AMPK complexes (Fig. 2, *D–F*). The data, together with the retention of carbohydrate binding for the phosphorylated KD mutant, underline that phosphorylation of AMPK at Thr-172 is compatible with its binding capacity to carbohydrates. Further, the binding of AMPK to β-cyclodextrin prior to activation is protective against the loss of binding ability.

We speculated that autophosphorylation could be responsible for the loss of binding to carbohydrates, because autophosphorylation is a consequence of the initial activation of kinases. Moreover, autophosphorylation at multiple sites has been demonstrated for AMPK upon activation (35, 36). Experimentally, it is difficult to initiate autophosphorylation without kinase activation. However, presuming a bimolecular mechanism, it is possible to test whether the phosphorylation of inactive AMPK by its active counterpart would result in the loss of carbohydrate-binding ability. Hence, inactive recombinant AMPK (α1β1γ1, α1β2γ1, or α1β1-ΔCBMγ1, respectively) was incubated with a very low amount of active AMPK (100:1 ratio). In the absence of ATP, both full-length AMPK proteins retained their ability to bind to the β-cyclodextrin resin, whereas the heterotrimer lacking the CBM was not capable of binding (Fig. 2*G*). However, in the presence of ATP, both AMPK isoforms were found in the supernatant fraction, indicating their loss of binding ability to the β-cyclodextrin resin despite the lack of Thr-172 phosphorylation by upstream kinases. The presence of the small fraction of active AMPK was confirmed by prolonged exposure (Fig. 2*G*, *bottom*). These results show that a catalytic amount of enzymatically active AMPK is sufficient to prevent inactive AMPK from binding to β-cyclodextrin in the presence of ATP, suggesting that a bimolecular autophosphorylation event precludes AMPK from β-cyclodextrin binding.

##### Autophosphorylation of AMPK at βThr-148 Causes a Loss of Binding Ability to Carbohydrate

**FIGURE 3.**
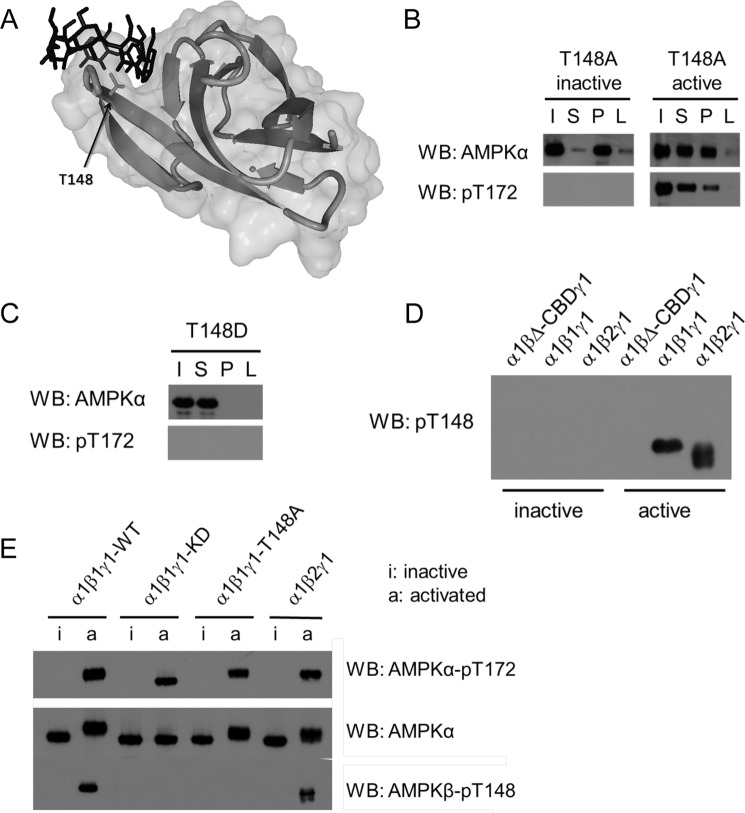
**Autophosphorylation of AMPK at βThr-148 causes a loss of carbohydrate-binding ability.** *A*, representation of AMPKβ1-CBM co-crystallized with β-cyclodextrin (Protein Data Bank code 1Z0M). The secondary structure of the protein is represented in a *light gray ribbon* and *molecular surface*, the β-cyclodextrin is shown in *dark sticks*, and the side chain of Thr-148 within the carbohydrate-binding pocket is highlighted in *sticks*. This image was generated using the software YASARA View (59). *B*, binding capacity of inactive and active AMPK bearing a non-phosphorylatable mutation at β1Thr-148 (T148A). *C*, effect of a phospho-mimicking modification of β1Thr-148 (T148D) on AMPK-carbohydrate binding. *D*, determination of Thr-148 phosphorylation on inactive and active recombinant AMPK complexes using a phospho-specific βThr-148 antibody. *E*, immunoblot analysis of inactive and active recombinant AMPK. WT AMPK or AMPK complexes bearing either a kinase-deficient (α1β1γ1-KD) (as in Fig. 1) or a non-phosphorylatable mutation at residue Thr-148 (T148A) were analyzed for Thr-148 phosphorylation using the phospho-specific Thr-148 antibody. AMPK presence was assessed using the total AMPKα antibody. Activation was determined by the Thr(P)-172 antibody. Data are representative of three experiments. *WB*, Western blot.

Considering that the binding of β-cyclodextrin prior to AMPK activation is protective from loss of binding, we speculated that the autophosphorylation event takes place directly in the carbohydrate-binding pocket of the CBM. Inspection of the x-ray structure of the β1-CBM identified Thr-148 as centrally located in the carbohydrate binding pocket (Fig. 3*A*). Using point mutagenesis, this site was mutated to an alanine (phosphorylation-resistant mutant T148A) in both inactive and active α1β1γ1 complexes. In the β-cyclodextrin binding assay, the inactive mutant conserved its binding capacity (Fig. 3*B*). However, in contrast to the active WT (Fig. 1*F*), the active T148A mutant partially conserved its carbohydrate-binding capacity upon co-expression of AMPK with the LKB1-Mo25-Stradα complex (Fig. 3*B*), implying a protective role of the T148A mutation.

To further establish the involvement of Thr-148 phosphorylation in the carbohydrate-binding ability of AMPK α1β1γ1, a phosphorylation-mimicking T148D mutant was incubated with the β-cyclodextrin resin. Although not being activated, the T148D mutant was unable to bind to the β-cyclodextrin resin (Fig. 3*C*). In addition, a phosphorylation site-specific Thr-148 (Thr(P)-148) antibody was developed, allowing for detection of the modification in AMPK by Western blotting. The recombinant activated WT AMPK α1β1γ1 and α1β2γ1 complexes showed a Thr-148 phospho-specific signal, whereas neither the inactive counterparts nor inactive/active α1β1ΔCBMγ1 showed detectable signals (Fig. 3*D*). Moreover, the Thr-172-phosphorylated KD and T148A mutants of AMPK α1β1γ1 did not show a Thr(P)-148 signal either (Fig. 3*E*), thus indicating the specificity of the Thr(P)-148 antibody. Therefore, the Thr-148 site is indeed autophosphorylated, and this modification directly correlates with the inability of AMPK to bind to the β-cyclodextrin resin. Taken together, these results indicate that autophosphorylation of AMPK at Thr-148 mediates its loss of carbohydrate-binding capacity.

##### The Phosphorylation-mimicking T148D Mutation Prevents AMPK from Binding to Glycogen

**FIGURE 4.**
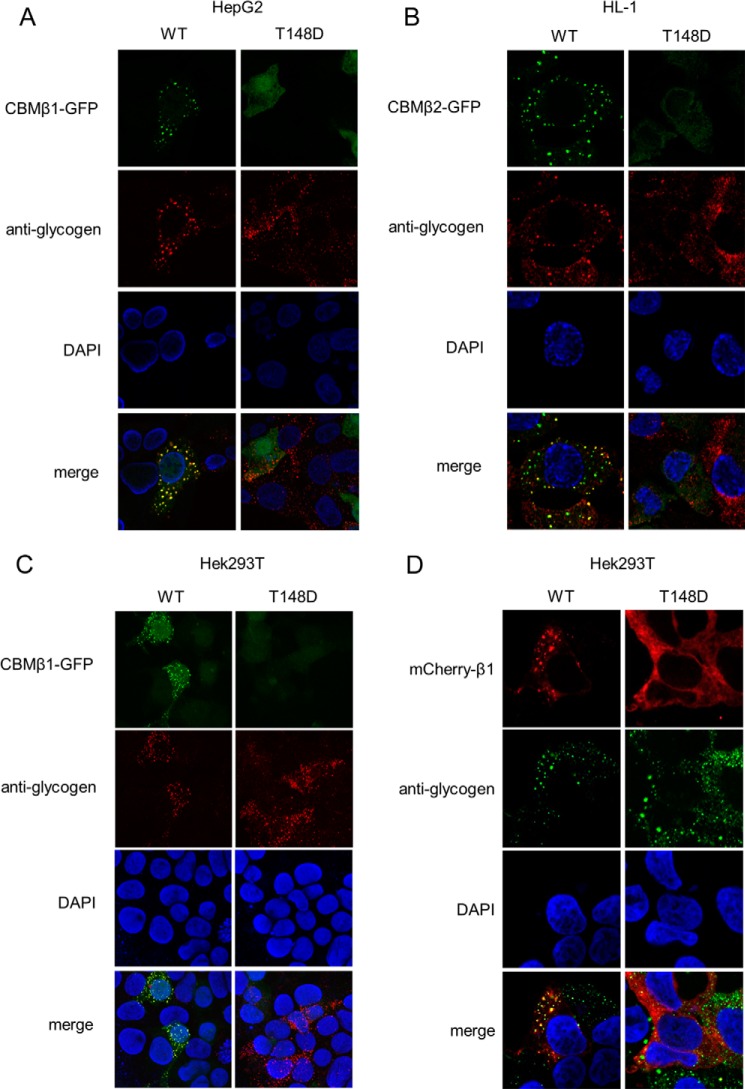
**The phosphorylation-mimicking AMPKβ-T148D mutation prevents AMPK from binding to cellular glycogen.** Shown are HepG2 (*A*), HL-1 (*B*), and HEK293T (*C*) cells stably overexpressing the WT and T148D mutant GFP-tagged β1-CBM or β2-CBM isoforms (in *green*), as indicated. Cells were fixed and stained with an anti-glycogen antibody that was detected by an Alexa647-labeled secondary antibody (in *red*) and with DAPI for nuclei (in *blue*), after which co-localization was assessed by confocal imaging. *D*, co-localization of WT or T148D mCherry-tagged β1-AMPK complexes (in *red*) was assessed as in *A* and *B* but using Alexa488-labeled secondary antibody for glycogen detection (in *green*). Data are representative of 3–5 experiments. Data have been adjusted for brightness and contrast to obtain best quality fluorescent images.

Next, we investigated whether the Thr-148 phospho-mimicking mutation (T148D) triggers a loss of binding of AMPK to glycogen, its natural ligand, both in the β1- and β2-isoforms. Our initial analysis concentrated on the isolated WT and T148D β-CBMs. Stable cell lines were established that expressed GFP-tagged CBM variants in the human hepatic cell line HepG2 and cultured mouse HL-1 cardiomyocytes. Immunofluorescence showed a marked speckled pattern of the WT GFP-tagged constructs, β1-CBM in HepG2 (Fig. 4*A*) and β2-CBM in HL-1 (Fig. 4*B*), both co-localizing with glycogen. In contrast, the β1- and β2-CBM T148D presented a diffuse pattern despite the presence of glycogen, as shown by a glycogen-specific antibody. Similar results were obtained using the HEK293T cell line stably expressing the β1-CBM (Fig. 4*C*); the speckled pattern of the WT CBM co-localized with glycogen, whereas the diffuse pattern of the mutant was incongruent with the observed glycogen staining. We further assessed the glycogen localization of mCherry-tagged full-length AMPKβ1-WT and T148D mutant after transient transfection of AMPK subunits in HEK293T cells. Results shown in Fig. 4*D* again revealed co-localization with glycogen for the WT, whereas the T148D mutant was diffusely located in the cytosol. Altogether, these findings indicate that the CBM of AMPK, expressed as an isolated domain or as a full-length protein forming part of the AMPK heterotrimer, naturally binds to glycogen, whereas the phospho-mimicking T148D mutation prevents this binding from occurring.

##### Endogenous AMPKβ-Thr-148 Phosphorylation Is a Dynamic and Regulated Process

**FIGURE 5.**
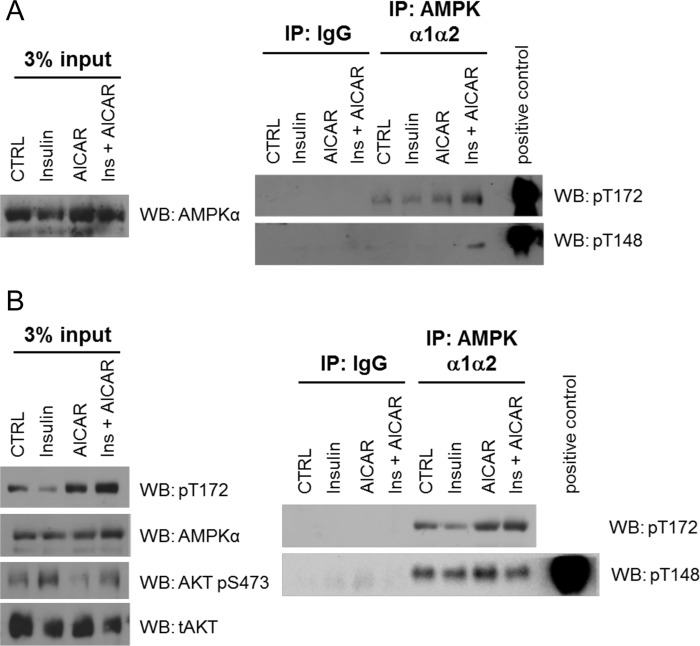
**Endogenous AMPKβ-Thr-148 phosphorylation is a dynamic and regulated process.** Shown is immunoprecipitation of endogenous AMPK with AMPKα1/α2 antibodies from HEK293T (*A*) or HepG2 (*B*) cells. Immunoblot analyses used total AMPKα, AMPKα-Thr(P)-172, AMPKβ-Thr(P)-148, total Akt, and Akt-Ser(P)-473 antibodies. Phosphorylation of AMPKβ-Thr-148 was detected following immunoprecipitation upon single treatment of insulin (100 nm, 15 min) or AICAR (1 mm, 45 min) or a combined treatment (*Ins* + *AICAR*). All immunocomplexes were assessed for activation of AMPK and βThr-148 phosphorylation using the Thr(P)-172 and Thr(P)-148 antibody, respectively. Data are representative of two experiments. *IP*, immunoprecipitation; *WB*, Western blot.

In order to investigate the occurrence of Thr-148 phosphorylation in cells and its detection with the Thr(P)-148 antibody, we immunoprecipitated endogenous AMPK from treated and untreated HEK293T or HepG2 cells (Fig. 5, *A* and *B*). Importantly, phosphorylation of Thr-148 could be detected in both cell lines, although under different conditions. In HEK293T, only the treatment with AICAR plus insulin led to the detection of the Thr-148 phosphorylation (Fig. 5*A*), and this treatment also showed the highest level of Thr-172 phosphorylation. In HepG2, however, Thr-148 phosphorylation occurred independently of AMPK activation changes (Fig. 5*B*). Hence, the data verify Thr-148 phosphorylation as an endogenous posttranslational modification. In addition, the differences of Thr-148 phosphorylation between cell types and conditions underline the control and dynamics of this post-translational modification.

##### Glucose Deprivation Triggers AMPKβ-Thr-148 Phosphorylation

**FIGURE 6.**
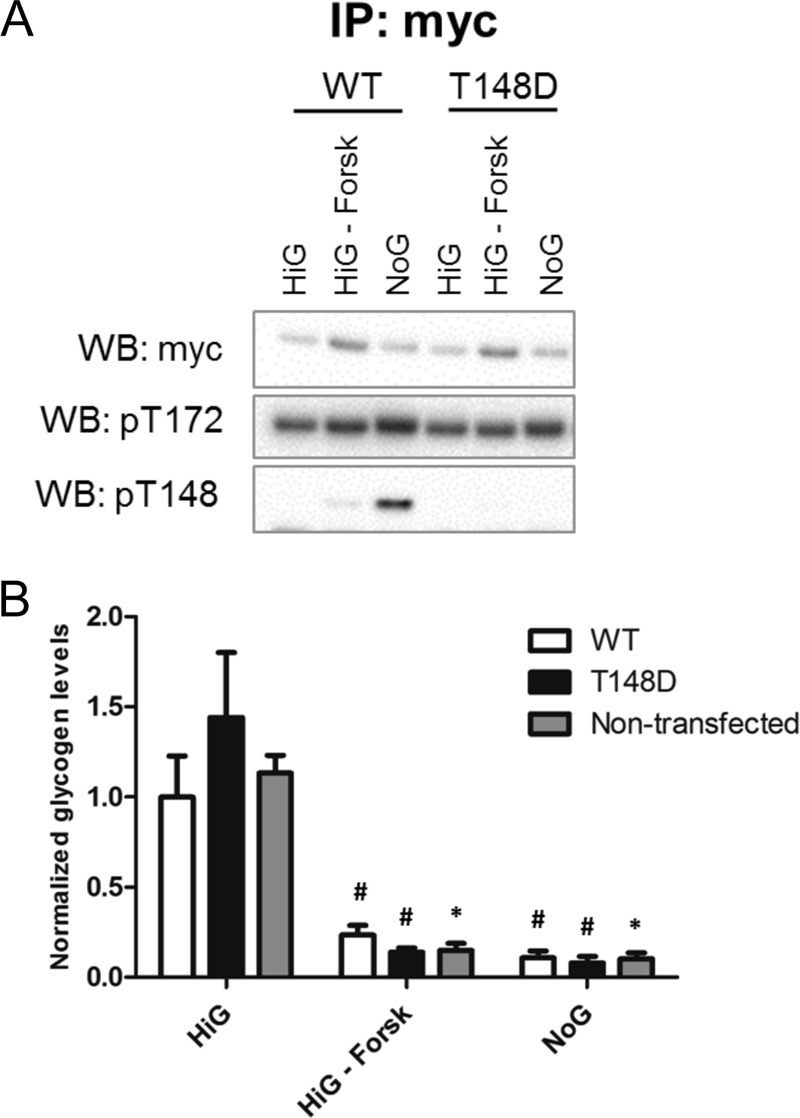
**Glucose deprivation triggers AMPKβ-Thr-148 phosphorylation.** *A* and *B*, HEK293T cells transiently overexpressing Myc-tagged α1, γ1, and WT or T148D mCherry-tagged β1 were treated for 16 h either with high glucose medium containing serum (*HiG*), with the same medium supplemented with forskolin (100 μm; *HiG-Forsk*), or with medium containing serum but no glucose (*NoG*). *A*, AMPKα was immunoprecipitated using the Myc tag antibody. Immunoblot using Myc tag antibody following precipitation shows the immunoprecipitation efficiency. Immunoblot with AMPKα-Thr(P)-172 and AMPKβ-Thr(P)-148 shows AMPK activity and autophosphorylation status. *B*, glycogen was quantified biochemically, corrected for protein concentration, and normalized to the level of WT cells under high glucose medium containing serum. Student's *t* test was used; treatment with high glucose medium containing serum *versus* treatment within one cell type (*, *p* < 0.05; #, *p* = 0.06) (*n* = 2). *IP*, immunoprecipitation; *WB*, Western blot.

To investigate the signals leading to AMPK Thr-148 autophosphorylation, we tested whether glycogen depletion induced AMPKβ-Thr-148 phosphorylation (Fig. 6*A*) and compared β1-WT- with β1-T148D mutant AMPK-expressing cells (Fig. 6*B*). HEK293T cells were either triple-transfected (as before) with AMPK γ1, Myc-tagged AMPKα1, and mCherry-tagged AMPKβ1 (WT or T148D) or left untransfected and then treated for 16 h with a high glucose medium with or without forskolin, a drug that promotes glycogen degradation (37), or with a medium without glucose. As expected, in forskolin-treated or glucose-deprived cells, the glycogen content was diminished if compared with control conditions in high glucose medium, although statistical significance was only reached in untransfected cells (Fig. 6*B*). In all of the three culturing conditions, the glycogen content was similar in untransfected, β1-WT, and β1-T148D mutant-expressing cells. Notably, Thr-148 phosphorylation was specifically induced by glucose deprivation but not forskolin, although both treatments depleted from glycogen (Fig. 6*A*). The Thr(P)-148 signal was absent in immunoprecipitates of T148D cells, reassuring that the antibody specifically recognizes the phosphorylation modification on the Thr-148 residue. Given the fact that increased AMPK activation by glucose deprivation is correlated with increased phosphorylation of Thr-148, these data support the cellular AMPK-mediated Thr-148 autophosphorylation mechanism.

##### βThr-148 Phosphorylation Plays a Role in Regulation of Glycogen Metabolism

**FIGURE 7.**
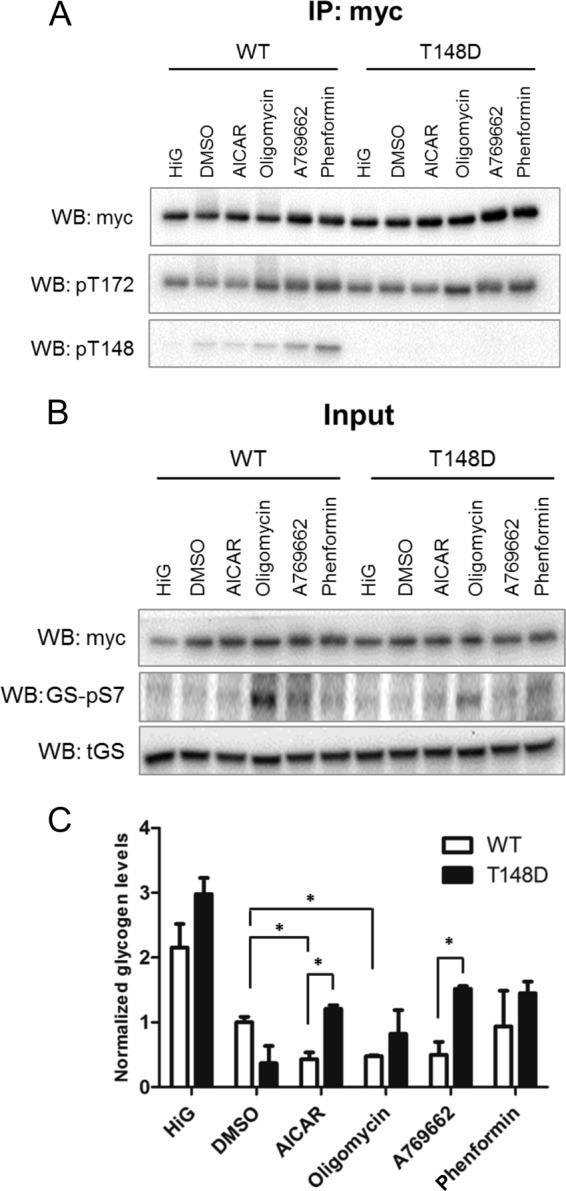
**AMPKβ-Thr-148 phosphorylation plays a role in regulation of glycogen metabolism.** *A–C*, HEK293T cells transiently overexpressing Myc-tagged α1, γ1, and β1-mCherry, either WT or β1 carrying the phospho-mimicking T148D (T148D), were serum-starved for 16 h prior to treatment with either high glucose medium (*HiG*) or DMSO, AICAR (1.5 mm), oligomycin (3 μm), A769662 (100 μm), or phenformin (1.5 mm) for 1 h. *A*, AMPKα was immunoprecipitated using the Myc tag antibody. Immunoblot using Myc tag antibody following precipitation shows the immunoprecipitation efficiency. Immunoblot with AMPKα-Thr(P)-172 and AMPKβ-Thr(P)-148 shows AMPK activity and autophosphorylation status. *B*, immunoblot of the associated input samples shows the expression levels of overexpressed Myc-protein and of glycogen synthase (*tGS*) and the phosphorylation status of glycogen synthase (*GS-pS7*). *C*, glycogen was quantified biochemically, corrected for protein concentration, and normalized to the level of WT cells under HiG. Student's *t* test was used (*, *p* < 0.05). *IP*, immunoprecipitation; *WB*, Western blot.

To further explore the physiological significance of Thr-148 phosphorylation, we treated cells with different AMPK activators and measured the resulting glycogen content. To allow for comparison of β1-WT with β1-T148D mutant AMPK, we again employed the HEK293T triple transfection model. As expected, in serum-starved cells, the treatment with oligomycin, A769662, and phenformin resulted in activation of AMPK in β1-WT as well as in β1-T148D cells, as observed by an increase in phosphorylation of Thr-172 (Fig. 7*A*). Further, the Thr(P)172 levels were paralleled by similar increases in Thr-148 phosphorylation with the Thr(P)-148 signal virtually absent in high glucose-treated cells and highest upon phenformin treatment. Cellular glycogen content generally decreased upon AMPK activation in accordance with the expected AMPK-induced shift toward catabolism (Fig. 7*C*). Statistically significant reductions were observed between vehicle and AICAR or oligomycin-treated WT-transfected cells. Interestingly, in A769662 or AICAR-treated cells, glycogen content was significantly higher in T148D *versus* WT-transfected cells, which may relate to AMPKβ1-T148D being absent from glycogen and thus unable to inhibit glycogen synthesis. Indeed, the AMPK-dependent inhibition of glycogen synthase (*GS*) by phosphorylation at serine 7 (*pS7*) was strongly increased in WT-transfected cells upon oligomycin and A769662, whereas the respective Ser(P)-7 signals were fainter in the T148D-transfected cells (Fig. 7*B*). Moreover, the glycogen content of WT-transfected cells treated with phenformin showed a difference neither with the vehicle-treated WT cells nor with the phenformin-treated T148D cells (Fig. 7*C*). Interestingly, this absence of difference correlated with the highest Thr-148 phosphorylation (Fig. 7*A*), suggesting that the autophosphorylation was sufficient to mimic the effect of the mutation. Taken together, Thr-148 is phosphorylated upon AMPK activation and thus prevents AMPK from binding to glycogen, which affects glycogen metabolism.

## DISCUSSION

In this study, we investigated the molecular mechanism causing AMPK to refrain from binding to glycogen, thereby affecting glycogen turnover. Our results demonstrate that bimolecular AMPK autophosphorylation at β-subunit Thr-148 interferes with its carbohydrate binding capacity. The Thr-148 site is located within the center of the carbohydrate-binding pocket, thereby controlling the attachment to glycogen. Accordingly, autophosphorylation at Thr-148 is prevented by carbohydrate occupancy. We also provide evidence that WT isolated CBMs and full-length β1-subunit as part of the AMPK heterotrimer localize to glycogen particles, whereas the respective T148D mutants do not bind cellular glycogen. In addition, we show that activation of AMPK enhances cellular Thr-148 phosphorylation and provide first evidence for involvement of β1Thr-148 phosphorylation in preventing AMPK from glycogen metabolism regulation.

It is established that many protein kinases catalyze their own activation by autophosphorylation (38). Autophosphorylation of AMPK has been described previously (11, 35, 36), but at present, little is known about the biological functions of AMPK autophosphorylation. Autophosphorylation of Ser-108 is required for activation of AMPK by the small molecule A-769662 independently of αThr-172 phosphorylation (39), but the physiological function of this modification has remained elusive so far. The phosphorylation of Ser-24/25 in the β1 subunit leads to nuclear exclusion (20), and Ser-24 and −25 were identified as autophosphorylation sites (35, 36). Here, we identify Thr-148 as a new autophosphorylation site and also ascribe its function to regulation of subcellular localization. Likewise, Feng and Hannun (40) reported a dissociation of protein kinase C (PKC) from the plasma membrane to the cytosol upon autophosphorylation. Also, the kinase ERK1/2 translocates to the nucleus upon autophosphorylation induced by extracellular signal, where it phosphorylates nuclear targets that are known to initiate cardiac hypertrophy (41). Hence, autophosphorylation, which more commonly has been regarded as a “mistake” of active kinases, rather emerges as a self-regulatory mechanism with possible consequences in subcellular targeting and is thus relevant for health and disease.

Our investigation revealed that little amounts of active AMPK were sufficient to prevent catalytically inactive AMPK from binding to glycogen, suggesting that a bimolecular autophosphorylation event is involved in this process. Therefore, it seems possible that few activated AMPK molecules are able to prevent glycogen binding of the entire pool of AMPK molecules, which would explain why the correlation of Thr-172 and Thr-148 phosphorylation was not always seen in cells. However, we can expect that the cell will have preventive measures, such as dephosphorylation of Thr-148, in order to restore glycogen binding. The phosphatase responsible for dephosphorylation therefore awaits identification.

Although some proteins can indirectly associate with glycogen via interacting with other glycogen-binding proteins (42), most proteins capable of directly attaching to glycogen or other polysaccharides possess a CBM. Regulation of glycogen binding by post-translational modifications has been reported previously. Notably, protein phosphatase 1 was found to detach from glycogen upon phosphorylation of its CBM-containing regulatory subunit G (43). Later work showed that this phosphorylation led to dissociation of protein phosphatase 1 from the G-subunit and its translocation to cytosol, whereas the G-subunit stayed bound to glycogen (44). Glycogen synthase, which does not carry a CBM, has also been reported to change cellular localization upon phosphorylation; when phosphorylated at site 1b, it associates with intramyofibrillar glycogen particles, whereas the site 2 + 2a phosphorylated enzyme binds to intermyofibrillar particles (45). Nevertheless, both of these phosphorylation events are unrelated to the glycogen-binding site of the glycogen synthase (18). AMPK carries a β-subunit CBM that is known to target proteins to glycogen (5, 6), but regulation of its glycogen-binding function is unknown because AMPK is found in various subcellular compartments, including those devoid of glycogen. In this current study, we provide evidence for a direct modification within the CBM, which we believe is the first example of such modification within a CBM resulting in loss of carbohydrate-binding affinity. We report the blocking of AMPK glycogen binding by Thr-148 autophosphorylation. Indeed, x-ray structural data indicated that Thr-148 of the AMPKβ subunit is positioned in the center of the carbohydrate-binding pocket and thus could be predicted to cause loss of binding upon phosphorylation. In support, our results revealed that the phosphorylation mimicking AMPK-T148D mutant was indeed incapable of binding to carbohydrates, suggesting that Thr-148 phosphorylation plays a role in localizing AMPK away from carbohydrates. This novel mechanism of AMPK regulation is furthermore consistent with studies using large scale phosphoproteome analyses and reporting Thr-148 as a β2 phosphorylation site (46, 47). Notably, in our study, we find that the β1- and β2-subunits are both modified by autophosphorylation at the Thr-148 site. Although the antibody was raised against the β2-sequence, it recognizes the modification in both isoforms (Fig. 3*E*). Therefore, based on our data, we cannot draw conclusions on isoform-specific differences of cellular Thr-148 phosphorylation.

To further evaluate the significance of Thr-148, we addressed the question of whether βThr-148 is being phosphorylated in cellular models. Our data showed that the levels of Thr-148 phosphorylation were mostly correlated with AMPK activity. In fact, based on our findings, we can assume that the molecular Thr-148 autophosphorylation mechanism we describe here and binding to glycogen occur in a mutually exclusive manner. In other words, AMPK-carbohydrate binding results in masking of the Thr-148 residue, which subsequently prevents AMPK from autophosphorylation. In line with this, the binding of β-cyclodextrin prior to activation was protective for the loss of carbohydrate binding ability. Moreover, insulin treatment prevented further increases of AICAR-mediated Thr-148 phosphorylation in HepG2 (Fig. 5*B*). This observation could be related to the fact that insulin promotes glycogen synthesis (48), thereby stimulating AMPK binding to glycogen and preventing it from autophosphorylation at Thr-148. As a possible interpretation of our results, even if AMPK in response to stresses would become very highly activated, Thr-148-phosphorylated, and, thus, prevented from interacting with glycogen, the glycogen-residing part of the AMPK pool would remain bound to carry on with AMPK activities at glycogen.

The precise roles of AMPK associated with glycogen are still subject to debate. On the one hand, AMPK stimulates GLUT4-mediated myocellular glucose uptake (49, 50) and co-immunoprecipitates with glycogen-binding proteins, such as glycogen phosphorylase (51), glycogen debranching enzyme (52), and glycogen synthase (53), suggesting that AMPK plays a role in the disposal of glucose into glycogen. In support of this notion, the phosphorylation of glycogen synthase by AMPK switches off the synthesis of glycogen (17, 54), and that of laforin reduces phosphatase activity and regulates its interaction with malin, thereby playing a major role in glycogen metabolism (55). However, on the other hand, Hunter *et al.* (53) delimited the supposed role of AMPK on glycogen synthesis by showing that the AMPK-mediated inactivation of glycogen synthase can be overridden by increased glucose 6-phosphate levels. Partly in line with this, McBride *et al.* (56) showed a clear inhibition of AMPK when bound to certain branch points of glycogen, raising doubt about the ability of AMPK to quickly phosphorylate its glycogen-bound downstream targets. Recently, Li *et al.* (57) showed that binding of the CBM to carbohydrates destabilizes the CBM-kinase domain interaction, thereby increasing the accessibility of the kinase domain for Thr-172 dephosphorylation, which is consistent with an inhibitory effect of glycogen-binding on AMPK kinase activity. Our findings suggest the irrelevance of AMPK glycogen localization for the process of glycogen degradation (Fig. 6). In contrast, the increased glycogen content in β1-T148D mutant- *versus* β1-WT-expressing cells upon AMPK activation (Fig. 7*C*) could be interpreted as an augmented rate of glycogenesis due to loss of AMPK glycogen localization and thus exclusion from glycogen synthesis inhibition. In any case, the unraveling of a new mechanism allowing for AMPK to stay unbound in the cytosol has consequences for our understanding of the kinase function, especially in the myocellular cell types, where glycogen is a major fuel resource (58). Hence, future efforts will be focused on determining the biological roles of βThr-148 phosphorylation in cardiac or skeletal muscle.

In summary, using cell-free systems and cellular models, we shed light on a novel molecular mechanism that is responsible for the loss of AMPK-glycogen interaction. Thr-148 autophosphorylation blocks the carbohydrate-binding pocket of the β-CBM, thus adding a new layer of complexity and identifying an unexpected switch regulating AMPK subcellular localization with relevance for glycogen metabolism.

## Footnotes

4 The abbreviations used are:

CBM: carbohydrate-binding module
AMPK: AMP-activated protein kinase
AMPKβ: β-subunit of AMPK
β1-CBM and β2-CBM: CBM of AMPKβ1 and -β2 subunit, respectively
iFCS: heat-inactivated fetal calf serum
LKB1: liver kinase B1
CaMKK2: calcium/calmodulin-dependent protein kinase 2
T148D: phosphorylation-mimicking mutation of AMPKβ Thr-148 residue to aspartate
T148A: phosphorylation-resistant mutation of AMPKβ Thr-148 residue to alanine
KD: kinase-deficient
AICAR: 5-aminoimidazole-4-carboxamide riboside
I, S, P, and L: input, supernatant, pellet, and Laemmli fractions of the β-cyclodextrin assay, respectively (see “Experimental Procedures” for more details).
